# Identification of CD4^+^ T Cell Epitopes in *C. burnetii* Antigens Targeted by Antibody Responses

**DOI:** 10.1371/journal.pone.0017712

**Published:** 2011-03-15

**Authors:** Chen Chen, Courtney Dow, Peng Wang, John Sidney, Amanda Read, Allen Harmsen, James E. Samuel, Bjoern Peters

**Affiliations:** 1 Department of Microbial and Molecular Pathogenesis, Texas A&M Health Science Center, College Station, Texas, United States of America; 2 Division of Vaccine Discovery, La Jolla Institute for Allergy and Immunology (LIAI), La Jolla, California, United States of America; 3 Department of Veterinary Molecular Biology, Montana State University, Bozeman, Montana, United States of America; Tulane University, United States of America

## Abstract

*Coxiella burnetii* is an obligate intracellular Gram-negative bacterium that causes acute Q fever and chronic infections in humans. A killed, whole cell vaccine is efficacious, but vaccination can result in severe local or systemic adverse reactions. Although T cell responses are considered pivotal for vaccine derived protective immunity, the epitope targets of CD4^+^ T cell responses in *C. burnetii* vaccination have not been elucidated. Since mapping CD4^+^ epitopes in a genome with over 2,000 ORFs is resource intensive, we focused on 7 antigens that were known to be targeted by antibody responses. 117 candidate peptides were selected from these antigens based on bioinformatics predictions of binding to the murine MHC class II molecule H-2 IA^b^. We screened these peptides for recognition by IFN-γ producing CD4^+^ T cell in phase I *C. burnetii* whole cell vaccine (PI-WCV) vaccinated C57BL/6 mice and identified 8 distinct epitopes from four different proteins. The identified epitope targets account for 8% of the total vaccination induced IFN-γ producing CD4^+^ T cells. Given that less than 0.4% of the antigens contained in *C. burnetii* were screened, this suggests that prioritizing antigens targeted by antibody responses is an efficient strategy to identify at least a subset of CD4^+^ targets in large pathogens. Finally, we examined the nature of linkage between CD4^+^ T cell and antibody responses in PI-WCV vaccinated mice. We found a surprisingly non-uniform pattern in the help provided by epitope specific CD4^+^ T cells for antibody production, which can be specific for the epitope source antigen as well as non-specific. This suggests that a complete map of CD4^+^ response targets in PI-WCV vaccinated mice will likely include antigens against which no antibody responses are made.

## Introduction

*Coxiella burnetii* is an obligate intracellular bacterium that causes Q fever in humans and animals. It is highly infectious and causes a wide variety of disease manifestations in humans as asymptomatic, acute and chronic forms [1], [2]. An effective formalin killed whole cell vaccine (Q-Vax®), produced from the phase I Henzerling strain of *C. burnetii*, has been administered to individuals that are skin test and serologically negative in Australia [3], [4], but no licensed vaccine exists in the US. Unfortunately, vaccination can result in severe local or systemic adverse reactions when administered to previously infected populations, and repeat vaccination can induce severe persistent reactions [4], [5]. To develop safe and effective new vaccines, it is therefore important to understand protective immune responses against *C. burnetii* that are induced by existing vaccines.

Several lines of evidence suggest that T cell dependent immune responses, especially CD4^+^ T cells, are induced by *C. burnetii* vaccines, and play a critical role in protective immunity against infection. Adoptive transfer of T cells from mice immunized with inactivated phase I *C. burnetii* whole cell vaccine (PI-WCV) was shown to provide protection for recipient mice [6], confirming the results from early studies [7], [8]. The fact that a major part of the vaccine-derived humoral response consists of IgG antibodies directed against proteins [9], [10] indicates the presence of relevant helper CD4^+^ T cell responses. Finally, low dose *C. burnetii* infection causes death in SCID and T cell deficient mice, but does not show a phenotype in B cell deficient mice, suggesting that T cells are essential for host resistance to *C. burnetii* infection [11].

Antigen specific CD4^+^ T cells generate protective immunity through different mechanisms: 1) the provision of cognate help to B cells, a requisite event for immunoglobulin (Ig) switching and affinity maturation in B cells [12], [13], 2) control of CD8^+^ T cells expansion and death, which appears to be essential for long-term CD8 memory responses [14], [15], and 3) direct secretion of cytokines including IFN-γ and TNF-α. The latter has been shown to be critical for intracellular bacterial clearance in general, and for *C. burnetii* in particular [11], [16], [17], [18], [19]. IFN-γ stimulates the production of nitric oxide and reactive oxygen molecules in macrophages, which are responsible for controlling infection [20], [21]. Treatment with IFN-γ may also induce *C. burnetii* killing by restoring the ability of the phagosome to mature and by promoting apoptosis of infected monocytes [22]. Consequently, IFN-γ has been successfully tested to treat chronic Q fever in a patient not responding to antibiotic treatment [23]. Inducing CD4^+^ T cells as a major source of IFN-γ is therefore highly desirable for vaccine-derived protective immunity.

The specific molecular targets of CD4^+^ T cell responses induced by *C. burnetii* PI-WCV vaccination are not known. Their identification could help guide which antigens to include in a new subunit vaccine, help develop correlates of protection for cellular immunity, and allow the use of tetramer reagents to elucidate the phenotypes and role of vaccine induced CD4^+^ T cells. Unfortunately, identifying targets of CD4^+^ responses is not trivial for bacterial pathogens for at least two reasons. The number of proteins encoded in the *C. burnetii* genome is large and most of them are thought to be included in the whole cell vaccine. This means a large number of potential targets needs to be included in the screen. Equally important, the frequency of responding CD4^+^ cells for a particular target protein is typically low. This means a strong stimulus and/or a highly sensitive assay is needed to reliably detect a response.

Previous studies that successfully identified targets of CD4^+^ responses in complex pathogens such as Mycobacterium and Salmonella [24], [25], [26] have focused on antigens targeted by B cell response. However, the success of this approach seemed to be at odds with classical studies of CD4^+^ responses that showed no linkage between antigens recognized by CD4^+^ and B cell responses, but rather a predominance of ‘intermolecular help’ [27], [28], [29], [30]. This apparent contradiction may be explained by a recent study that found a strong correlation between CD4^+^ and antibody responses on the antigen level in vaccinia virus infected mice [31]. The authors of that study suggested that classical investigations of linkage between CD4^+^ and B cell response targets might not have encountered this effect because they were examining small viral pathogens such as influenza or hepatitis B virus.

In this study, we utilized bioinformatic predictions to identify candidate peptide epitopes in a set of *C. burnetii* antigens that were previously shown to be targets of immunodominant B cell responses. We identified eight H-2 I-A^b^ restricted *C. burnetii* CD4^+^ T cell epitopes in four antigens which elicited high IFN-γ production following vaccination. This is the first set of T cell epitopes identified in *C. burnetii* and this strategy can guide future studies in subunit vaccine development against Q fever.

## Results

### Selection of a candidate set of targets for CD4^+^ responses following PI-WCV vaccination

**Figure 1:**
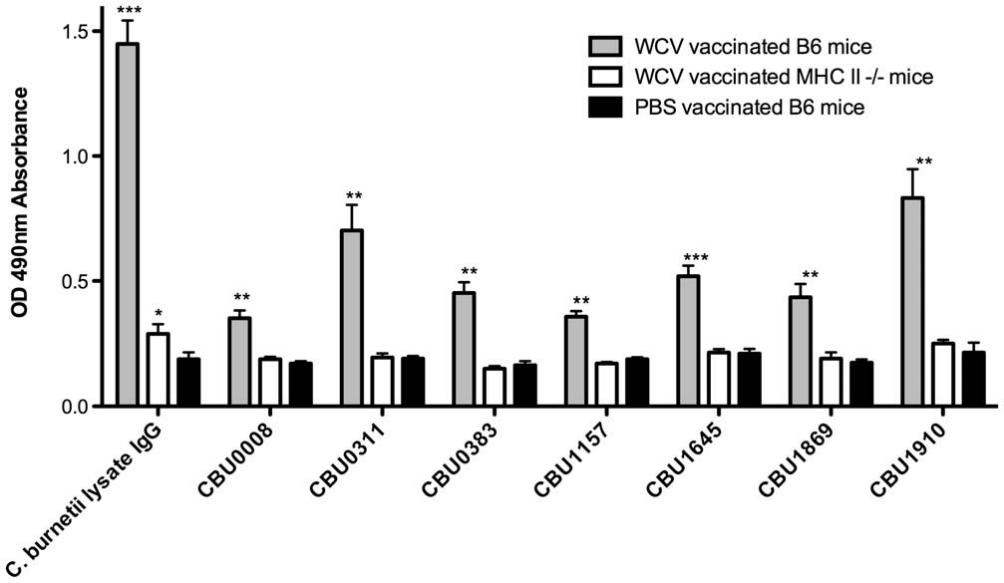
IgG antibody production for selected *C. burnetii* proteins is MHC-II dependent. Seven recombinant proteins were generated and tested by ELISA for IgG reactivity of PI-WCV vaccinated C57BL/6 mice, C57BL/6 MHCII deficient (*H2-Ab* ^−/−^) mice and PBS vaccinated C57BL/6 mice sera, 14-day post second vaccination. *C. burnetii* lysate was included as a positive control. PI-WCV vaccinated B6 mice generated detectable IgG responses against all antigens tested, while MHCII deficient mice showed no detectable IgG responses against individual recombinant proteins and only weak responses against *C. burnetii* lysate. *P<0.05, **P<0.01, ***P<0.001 comparing to PBS vaccinated B6 mice.

The humoral responses following *C. burnetii* infection and vaccination has been shown to be directed against both protein and glycolipid fractions of *C. burnetii* [32], [33], [34], [35]. The fact that a major part of the response consists of IgG antibodies directed against proteins [33], [35] indicates the presence of relevant CD4^+^ T helper cell response. Protein microarrays using a group of vaccinated and convalescent human sera have identified a set of *C. burnetii* immunodominant proteins [36]. Of these four immunodominant proteins (CBU 0008, CBU 0383, CUB1157, CBU 1869), two previously identified immunodominant proteins (CBU 0311 [37] CBU 1910 [32]) and one previously unpublished antigen (CBU 1645, personal observation) were selected (Table 1) and tested for recognition by antibodies in PI-WCV vaccinated C57BL/6 wild type mice (Figure 1). Prime and boost PI-WCV vaccination generated detectable IgG responses against all seven recombinant antigens in C57BL/6 wild type mice. Antibody responses were also measured in vaccinated C57BL/6 MHCII deficient (*H2-Ab* ^−/−^) mice, in which no significant IgG production was detected against any of the individual antigens. This confirms that IgG production against all tested proteins is MHCII dependent and provides further evidence for the presence of vaccine induced CD4^+^ T cells.

**Table 1:** Summary of antigen and peptide selection.

ORF ID	Protein Function^a^	Putative localization^b^	Protein length (aa)	Number of predicted I-A^b^ binding peptides
CBU 0008	Hypothetical protein	Unknown	62	12
CBU 0311	Outer membrane porin	Outer membrane	223	29
CBU 0383	DNA-3-methyladenine glycosylase	Cytoplasmic	204	8
CBU 1157	Hypothetical exported lipoprotein	Cytoplasmic Membrane	233	12
CBU 1645	Type IV secretion DotB protein	Cytoplasmic	372	25
CBU 1869	Hypothetical exported protein	Unknown	217	8
CBU 1910	Outer membrane protein	Periplasmic/Outer membrane	252	23

a TIGR annotations.

b Predicted by PSORTb [56].

We next wanted to examine if the immunodominant B cell antigens in *C. burnetii* are also targets of CD4^+^ responses. We screened the sequences of the seven B cell antigens (Table 1) for peptides predicted to have high affinity for the MHC class II molecule H-2 IAb using a consensus prediction approach (see Materials and Methods and [38]). A set of 117 candidate peptide sequences, 15 residues in length, were selected from the 7 different antigens, and synthesized for further study.

### Identification of H2 I-Ab restricted epitopes in vaccinated C57BL/6 mice

In an initial screen, the antigenicity of the 117 candidate peptides was tested using cells from C57BL/6 mice obtained 10 days following s.c. vaccination with each recombinant protein in the context of complete Freud's adjuvant (CFA). Responses were evaluated in IFN-γ ELISPOT assay with CD4^+^ T cells purified from individually pooled spleen and lymph nodes of 5 mice. The number of spot forming cells (SFC) following peptide stimulation was recorded in triplicate along with media stimulated cells as negative controls. Any peptide yielding >20 mean net SFC/10^6^ cells above background and a Stimulation Index (SI = SFC peptide/SFC media control) >2 with a *p*<0.05 in a standard t test in two independent experiments was considered positive. A total of 20 positive peptides with SI values between a range of 2 and 19 were identified (Table S1). In contrast, none of the peptides elicited positive responses in naïve mice (data not shown).

Peptides that were positive in the initial screens were tested for recognition by CD4^+^ T cells purified from 6 C57BL/6 mice 10 days post s.c. vaccination of PI-WCV homogenized with incomplete Freud's adjuvant (IFA) and CpG. A total of 11 out of 20 originally identified peptides elicited positive responses following PI-WCV vaccination (>20 SFC/10^6^ cells, SI>2 with a *p*<0.05). This included three pairs of peptides that overlapped by 11 or more residues and therefore are likely recognized by the same T cells. For each of these pairs, the peptide with the higher response following WCV vaccination was chosen as the optimal representative, resulting in a total of 8 distinct epitopes from 4 different *C. burnetii* proteins (Table 2). The eight identified CD4^+^ epitopes are conserved among all sequenced *C. burnetii* strains, and we did not find any identical sequences in other bacteria species by BLAST.

**Table 2:** Summary of distinct CD4^+^ T cell epitopes recognized after vaccination with recombinant proteins and PI-WCV.

Peptide ID	Sequence	H-2 I-Ab binding affinity [IC50 nM]	IFN-γ T cell ELISPOT [SI]	
			after vaccination^a^	after infection^b^
CBU 0383_69–83_	RDSFNNFDASIISKY	507	5.3	4.4
CBU 1157_63–77_	PWRYIRSFPILASSG	34	6.8	4.2
CBU 1157_108–122_	LSLMLNYPNSADRYY	1040	4.8	-
CBU 1157_177–191_	DLRYHAPIYGAVHPR	193	3.7	3.8
CBU 1645_196–210_	YDSLTTPTASVCQSE	620	4.1	3.5
CBU 1645_272–286_	RLVGSFPAEERIGRT	1858	3.5	-
CBU 1910_45–59_	HYLVNHPEVLVEASQ	293	23.8	28.3
CBU 1910_83–97_	KLFNDPASPVAGNPH	351	3.7	3.8

a Mice were subcutaneously vaccinated with 10 ug PI-WCV/mouse(IFA+CpG) for 10 days. Peptide at 1 ug/ml. Three Independent experiment (n = 6). T cells were tested in ELISPOT assays for IFN-g production. Data is presented as Stimulation index (SI), calculated as the number of spot forming cells after peptide stimulation divided by spot forming cells in background. SI>2 was used as cut-off for positive response.

b Mice were intratracheally infected using 10^6^ *C. burnetii* RSA439 strain for 12 days. Peptide at 1 ug/ml. Three independent experiment (n = 4). ELISPOT assays were performed as for post-vaccination.

### Infection and vaccination induced similar epitope recognition hierarchy

We next wanted to determine if the epitopes identified in vaccinated mice overlap with those recognized in infected mice. All peptides listed in Table S1 were tested for recognition by purified CD4^+^ T cells derived from C. *burnetii* RSA 439 infected C57BL/6 mice. A total of 9 out of 20 originally identified peptides elicited positive responses following 10^6^ RSA 439 intratracheal infection (>20 SFC/10^6^ cells, SI>2 with a *p*<0.05). All 9 peptides were also recognized in PI-WCV vaccinated mice. By far the strongest response in both cases was directed against CBU 1910_45–59._ The next two highest responses were in both cases directed against CBU 0383_69–83_ and CBU 1157_63–77_. Only two peptides (CBU 1157_108–122_ and CBU 1645_272–286_) with low responses following vaccination did not meet the significance cutoff for recognition following infection (Table 2). Overall, these results indicate that C. *burnetii* vaccination and infection lead to essentially identical spectrum of epitopes recognized.

### Characterization of the H-2 I-Ab restricted epitope by ICCS

**Figure 2:**
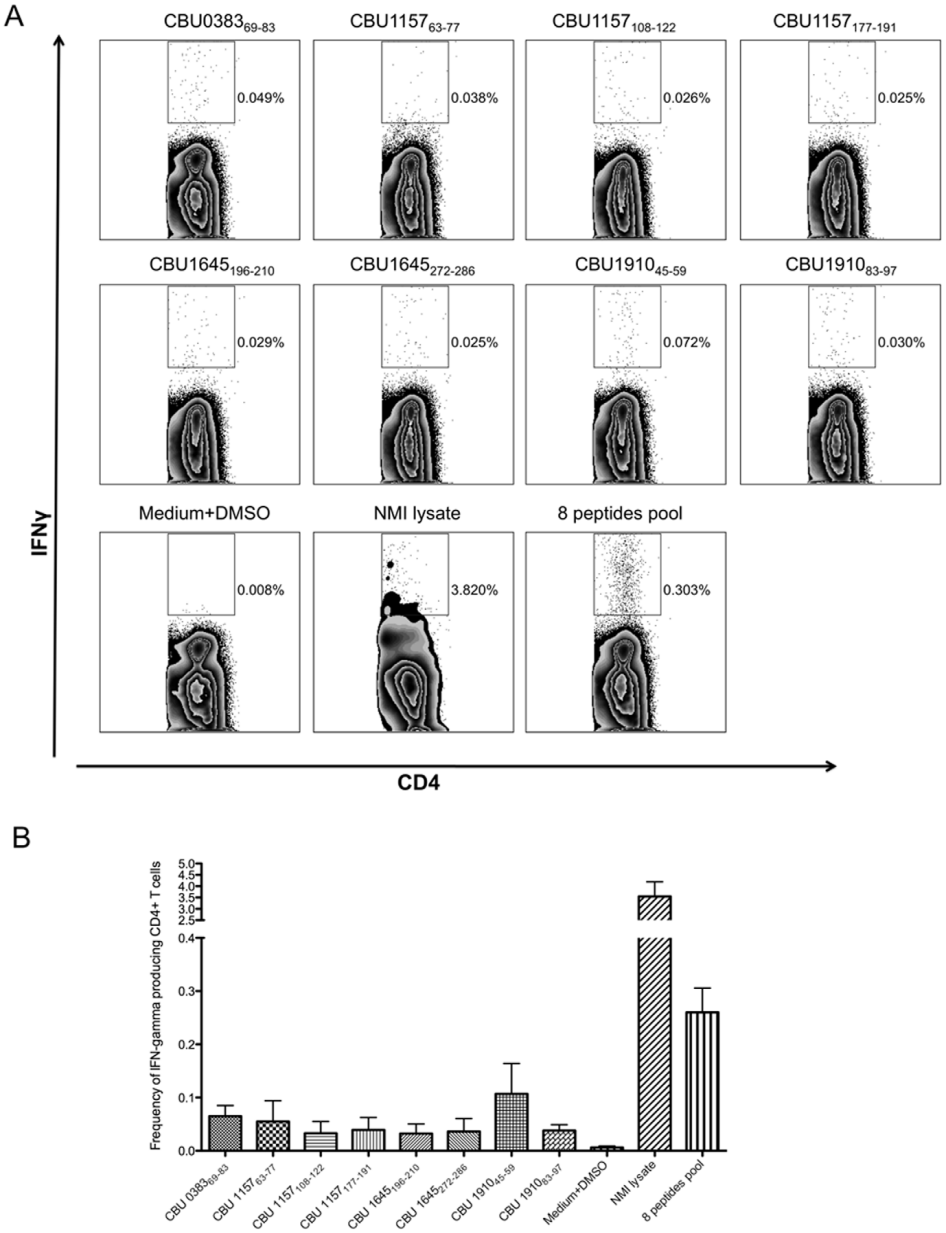
CD4^+^ T cells recognition of PI-WCV derived H-2 I-A^b^ epitopes in IFN-γ ICCS assays. CD4^+^ T cells recognition of positive peptides identified by IFN-γ ELISPOT were tested in ICCS assay. Ten ug of each peptide was used to stimulate 2×10^6^ lymphocytes from three mice immunized with PI-WCV in the context of IFA and CpG 10 days earlier. Pool 8 used a mixture of eight peptides, which represents 8 different epitopes. A representative experiment of four total experiments is shown in (A). Percentages of IFNγ producing CD4^+^ T cells following stimulation with lysed Nine Mile phase I *C. burnetii* and peptides are shown. A peptide was considered positive if the average of the individual experiments resulted in at least >1 SD above background (Medium +DMSO). Panel B shows the average frequency and standard deviation of epitope specific IFNγ producing CD4^+^ T cells from four-independent experiments.

We used an ICCS assay to obtain the frequency of peptide specific IFN-γ producing CD4^+^ T cells after PI-WCV vaccination. Figure 2 shows a representative experiment using the 8 optimal peptides that elicited IFN-γ production in both rAgs and PI-WCV immunized mice in ELISPOT assays. The results from four independent experiments are summarized in Table 2. The frequency of responding cells ranged from 0.03% to 0.11%. All peptides induced IFN-γ responses >1 SD above background cutoff (0.025%) and 5 of 8 peptides induced >2 SD above background (0.034%).

Next, we wanted to assess the fraction of the total response accounted for by the identified epitopes. As shown in Figure 2, 3.8% of the total CD4^+^ T cells from PI-WCV vaccinated mice produced IFN-γ upon stimulation with *C. burnetii* lysate (NM I lysate). A pool of the 8 optimal epitopes induced a response of 0.3%. Based on these data, the peptides identified represent targets of about 8% of the total specific IFN-γ^+^CD4^+^ response induced by PI-WCV vaccination.

Recent studies suggested that multifunctional, high-level cytokine-producing (IL-2, IFN-γ and TNF-α) Th1 cells are correlated with protection against many intracellular pathogen infections, including HIV, *Leishmania major* and *Mycobacterium tuberculosis* [39], [40]. In order to determine the quality of PI-WCV vaccination induced peptide specific CD4^+^ T cells, we performed an in depth analysis of peptides specific Th1 cells induced by PI-WCV vaccination. We found that all 8 epitopes can induce detectable IL-2^+^ CD4^+^ T cell recall responses in PI-WCV immunized mice (Figure S1). However, only two peptides (CBU 1157_63–77_ and CBU 1910_45–59_) induced detectable TNF-α^+^ CD4^+^ T cell recall responses in PI-WCV immunized mice (Figure S2). Multicolor analysis confirmed this observation that only a small fraction of CBU 1157_63–77_ and CBU 1910_45–59_ specific IFN-γ producing CD4^+^ T cells secret TNF-α in PI-WCV immunized mice. Due to the lack of TNF-α expression, none of PI-WCV vaccination induced peptides specific CD4^+^ T cells were capable of secreting high-level IL-2, IFN-γ and TNF-α simultaneously (Figure S3). Notably, only about 1.5% of T cells responding to PI-WCV lysate overall were multifunctional. This suggests that either multi-functional T cells are not required for the protective effect of PI-WCV vaccination, or that the low number of multifunctional T cells we observed can nevertheless contribute to protection.

### H-2 I-A^b^ binding capacity of identified epitopes

To determine the MHC binding affinity for each of the identified epitopes, we conducted MHC-peptide binding assays. H-2b class II MHC was purified and quantitative inhibition binding assays were performed for each peptide using IAb as described in Materials and Methods and [41]. The binding assays revealed that all of the identified peptides bound to IAb with significant affinities in the range of 34 – 1858 nM (Table 2). Six of the eight peptides bound with affinities higher than 1,000 nM. This is consistent with previous studies of class II binding which found that the majority of epitopes have affinities above this threshold [42]. Taken together, these data further characterize the epitopes identified and support their putative I-Ab restriction, assigned on the basis of the recognition by CD4^+^ T cells.

### Immunization with peptide CBU 0383_69–83_ did not protect from challenge

Immunization with the whole cell vaccine induces protective immunity from challenge with virulent *C. burnetii* organisms (45). We tested if immunization with a CD4^+^ epitope would induce a measurable protective effect. We chose peptide CBU 0383_69–83_ for these experiments as it induced one of the three strongest responses, and we had sufficient amounts of purified material available. Four groups of mice were immunized with either the epitope, heat killed *C. burnetii* as a positive control, PBS (sham) or an irrelevant CD4^+^ epitope (OVA), respectively. Body weights were recorded at 2–4 day intervals after infection with *C. burnetii* Nine Mile phase I through 14 days p.i. The mice were sacrificed and lungs removed for PCR analysis and spleens weighed. Examination of the body weights indicated that at days 7 and 10, mice immunized with the heat killed *C. burnetii* control lost significantly less weight than mice immunized with either OVA or the epitope (*p*<0.01) (Figure S4). However, no protective effect of the epitope immunization was detectable by either weight loss, spleen size on day 14, or bacterial burden in the lung detected by PCR (data not shown). We conclude that immunization with this peptide alone is insufficient to convey measurable protective immunity against respiratory *C. burnetii* infection. Additional experiments will be necessary to assess the protective capacity of the other identified epitopes and of epitope pools.

### Antigen specific linkage between CD4^+^ T cell help and antibody production

**Figure 3:**
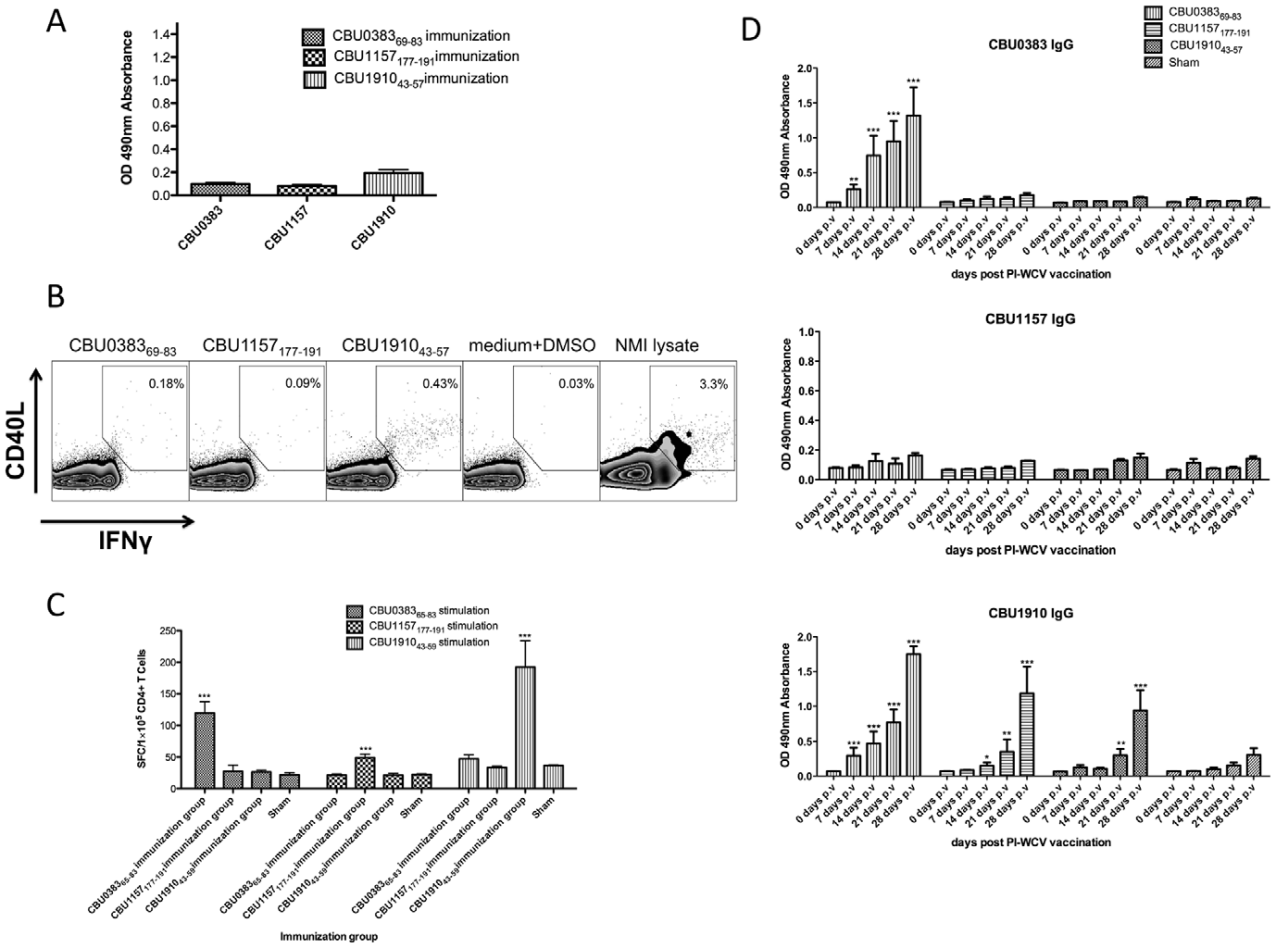
Selective protein-specific CD4^+^ T cell help to B cells after peptide and PI-WCV vaccination. Four group of C57BL/6 mice (n = 5) were primed with three *C. burnetii* CD4^+^ epitopes (CBU 0383_69–83_,CBU 1157_177–191_ and CBU 1910_43–57_) in the context of IFA and CpG or adjuvant alone, followed by PI-WCV vaccination 10 days later. (A) IgG antibody responses to three individual *C. burnetii* proteins after twice peptide vaccination. (B) At 28 days post PI-WCV vaccination, splenocytes from peptide and PI-WCV immunized mice were stimulated with peptides and lysed Nine Mile Phase I *C. burnetii* respectively, for 20 hours. Samples were subsequently stained for intracellular IFNγ and CD40L. Gated CD4^+^ lymphocytes are shown, and quantified percentages represent CD4^+^IFNγ^+^CD40L^+^ T cells. (C) Frequency of peptide specific IFN-γ producing CD4^+^ T cells at 28 days post PI-WCV vaccination. (D) IgG antibody responses to each *C. burnetii* protein at 0 days, 7days, 14 days, 21 days and 28 days post PI-WCV vaccination. *P<0.05, **P<0.01, ***P<0.001

Next, we wanted to explore what causes a significant fraction of CD4^+^ responses to target antigens that are also targeted by antibody responses. In particular, we wanted to test if T cells predominantly provide help for antibody responses against the source antigen of their target epitope. Such a functional correlation between antibody and CD4^+^ response targets was previously found in Vaccinia Virus [31]. In order to determine linkage between antibody production and CD4^+^ T cell specificity in PI-WCV vaccinated mice, C57BL/6 mice were immunized with representative CD4^+^ epitopes from three different antigens (CBU 0383_69–83_, CBU 1157_177–191_ and CBU 1910_43–57_) followed by PI-WCV vaccination 10 days later. Sera were collected each week after vaccination to measure IgG reactivity against the three corresponding proteins. Peptide immunization alone did not generate detectable antibodies against any tested recombinant proteins (Figure 3A), which indicates that these three peptides do not contain B cell epitopes that elicit antibody production against their source antigen. The frequency of epitope specific IFN-γ producing CD4^+^ T cells was measured at 28 days post PI-WCV vaccination (Figure 3B and 3C). All mice receiving peptide immunizations contained more peptide-specific CD4^+^ T helper cells compared to the mice in the sham immunization group, which received PI-WCV immunization without any peptide priming. This result demonstrates that epitope specific CD4^+^ T cells were successfully primed by peptide immunization.

The presence of CD4^+^ help for B cells producing antibodies against each of the three antigens was determined by comparing antibody titers with and without peptide immunization. Surprisingly, the pattern of help provided proved to be different for all three antigens (Figure 3D): Immunization with any of the three peptides provided nonspecific help for IgG antibody production against CBU 1910 by 21 days post PI-WCV vaccination compared to the sham control group. None of the three peptide immunizations improved anti-CBU 1157 IgG antibody production compared to the sham immunization control group. Finally, only peptide CBU 0383_69–83_ immunization provided specific help for anti-CBU 0383 IgG antibody production beginning 7 days post PI-WCV vaccination. These results suggest that undefined antigen specific factors influence whether a linkage between antibody and CD4^+^ T cell responses is established.

## Discussion

Several studies suggest that T cells, especially CD4^+^ T cells, play pivotal roles in vaccine derived protective immunity against *C. burnetii* infection. However, little is known about how CD4^+^ T cells are generated in response to *C. burnetii* PI-WCV vaccination and what their molecular targets are. One reason for this gap in knowledge is that traditional CD4^+^ T cell antigen screening and epitope mapping methods are hampered by the large genome size of bacteria including *C. burnetii*. Bioinformatics predictions of peptide:MHC binding have been successfully utilized to identify peptide candidates for epitope mapping [43]. However, the number of candidate peptides predicted for CD4^+^ epitopes in *C. burnetii* is in the order of several thousands. We examined whether a strong B cell response against an antigen is accompanied by a CD4^+^ T cell response, and if such a correlation could be utilized as a mapping strategy of CD4^+^ responses in *C. burnetii*. More than 20 immunodominant proteins, which have significantly elevated IgG antibody titer after infection or PI-WCV vaccination, have been identified and characterized in several studies [32], [33], [34], [35], [36]. Presumably, these proteins are preferably targeted by B cells because they are either abundant in *C. burnetii* or because B cells targeting them receive preferential CD4^+^ T cell help, which is required for B cell proliferation and Ig isotype switching. As expected, a comparison of PI-WCV generated antibodies in B6 and MHCII knock-out mice showed that generating IgG antibodies against these proteins required MHCII mediated CD4^+^ T cell help (Figure 1). Therefore we screened for CD4^+^ epitopes in proteins that are known to be targeted by B cell responses.

The applied B cell antigen-guided mapping strategy identified CD4^+^ T cell epitopes in *C. burnetii* with a similar success rate as was reported in viruses with about ∼10 fold smaller genomes. Using bioinformatic predictions, we identified a set of 117 predicted H-2 IA^b^ binding peptides in 7 proteins targeted by B cell responses. We screened these peptides for recognition in B6 mice vaccinated with PI-WCV, and identified 8 distinct *C. burnetii* CD4^+^ T cell epitopes. This hit rate (8/117 = 7%) compared favorably with a recent study mapping class II epitopes in Vaccinia Virus [43] which screened 2146 peptides and identified 14 epitopes (0.7% hit rate). In another recent study [44], 200 predicted high affinity binding peptides from MCMV were screened and 15 epitopes identified ( = 7.5% hit rate). This demonstrates that at least a subset of CD4^+^ epitopes can be efficiently identified by predictions of MHC class II binding peptides in proteins known to be targeted by B cell responses. The identified epitopes provide the first insights into the targets of CD4^+^ responses in *C. burnetii*. No responses were detected against the CBU 0008, CBU 0311 and CBU 1869 proteins, which suggests that these proteins are weakly immunogenic for CD4^+^ T cells in mice. Approximately one-half of the epitopes recognized following immunization with recombinant antigens were also recognized following PI-WCV vaccination and infection, which confirmed the immunogenic nature of these antigens. Among the 8 identified epitopes, the frequency of CBU 1910_45–59_ specific CD4^+^ T cells (Table 2 and Figure 2) was much higher than for other epitopes. Interestingly, CBU 1910 was also the protein against which the highest antibody titers were detected (Figure 1). Peptides from this antigen are now being examined for their potential to serve as a correlate of protection for *C. burnetii* vaccine efficacy in the murine system, and to assess the potential for developing an IFN-γ based immunodiagnostic assay, which could provide direction for more accurate human Q fever diagnosis.

We estimated the fraction of the total CD4^+^ response targets accounted for by the 8 identified epitopes by comparing to the response against whole *C. burnetii* extract. In FACS assays, 3.8% of CD4^+^ cells produced IFN-γ when stimulated by the extract. In comparison, a pool of the 8 peptides stimulated 0.3% of the CD4^+^ cells, which corresponded to about 8% of the extract response. While identification of only a small fraction of the total response was an expected limitation of our screening strategy, we aimed at identifying the most likely targets of responses rather than achieving complete coverage. A second limitation to our screen is that it only characterized IFN-γ producing T cells. We predicted that Th1 cells that secret IFN-γ are critical to confer PI-WCV derived protective immunity against *C. burnetii* infection. However, additional CD4^+^ T cell epitopes may exist in our original pool that do not induce IFN-γ, but do trigger production of other cytokines, such as IL-2, IL-4, IL-10 or TNF-α.

Earlier studies have reported deterministic linkages of antigen specificities [31]. For example, immunization with CD4^+^ epitopes provided specific help for B cells recognizing that antigen. While the mechanism behind this effect was not determined, the authors suggested that antigen uptake by B cells may not involve internalization of whole viral particles, resulting in selective antigen presentation by different B cells via MHC class II and selective help by CD4^+^ T cells. We tested if a similar linkage between antibody production and CD4^+^ T cell specificities was present in the epitopes identified in our study. Surprisingly, we found a diverse pattern of linkage for three epitopes from three proteins. Mice immunized with each of three peptides enhanced anti-CBU 1910 IgG antibody production after PI-WCV vaccination. Alternatively, only one peptide (CBU 0383_69–83_) induced CD4^+^ T cells that provided specific help for anti-CBU 0383 IgG production in PI-WCV vaccinated mice. None of the peptides induced help for anti-CBU 1157 IgG production. These data suggest that the linkage between CD4^+^ help and antibody responses in the PI-WCV vaccination model is complex and epitope specific.

We speculate that the reason for this complex linkage is that B cells utilize multiple mechanisms to sample *C. burnetii* antigens after PI-WCV vaccination which do not always involve the internalization of whole killed bacteria. Specifically, we propose two models. The first model proposes that two formats of antigens, soluble proteins and intact *C burnetii* bacteria, are recognized by B cells through different routes, which is well documented by several studies [45], [46], [47], [48], [49], [50], [51]. In this model, membrane associated proteins (like CBU 1910) are always recognized and internalized by B cells together with other bacterial components, and these antigen specific B cells in turn receive more nonspecific CD4^+^ T cell help for proliferation and IgG antibody production. In contrast, cytoplasmic proteins (like CBU 0383) that are processed individually by cognate B cells after being released by bacteria only receive help from CD4^+^ T cells specific for the same antigen. This model also explains our observation that PI-WCV vaccination can induce significantly more IgG antibody responses to membrane-associated proteins than soluble extraction of *C.burnetii* (data not shown). A second model proposes selective antigen presentation at the postendocytic level by more specific targeting mechanisms. Endocytosis of antigen is not necessarily sufficient to ensure efficient antigen presentation [52]. Different *C. burnetii* protein antigens may utilize different B cell surface receptor for oligomerization, which could affect sorting along the endocytic pathway. Different postendocytic sorting steps may affect T cell epitopes that are processed by B cells [52], [53]. Further studies are required to discriminate between these two mechanisms.

In conclusion, our data demonstrate the feasibility of using antibody responses and MHC class II binding predictions to identify targets of CD4^+^ T cell responses in PI-WCV vaccinated mice. Using some of these newly identified CD4^+^ epitopes, we conclude that B cells processing *Coxiella* may involve multiple, distinct mechanisms with an intricate linkage between antibody production and CD4^+^ T cell specificities. The fact that not all CD4^+^ epitopes provided help for IgG production against their source antigens suggests that antigens against which no IgG production can be detected may well harbor targets of CD4^+^ responses. Finally, eight newly identified CD4^+^ T cell epitopes will provide new reagents for tracking and analyzing the immune responses targeting *C. burnetii*.

## Materials and Methods

### Bacteria

*C. burnetii* Nine Mile phase I (RSA 493) and Nine Mile phase II (RSA 439) were grown in embryonated chicken eggs, purified by gradient centrifugation, and inactivated by electronic beam irradiation (32).

### Cloning and expression of recombinant proteins

Open reading frames corresponding to *C. burnetii* immunoreactive proteins were amplified by PCR and cloned into the pBAD/TOPO ThioFusion expression vector (Invitrogen, Carlsbad, CA). Recombinant proteins were expressed as 6×His-tagged fusion proteins in *E. coli* Top 10 and purified by nickel affinity chromatography (Invitrogen).

### CD4^+^ T cell epitope prediction and peptides synthesis

Seven protein antigens were selected for T cell epitopes mapping; CBU 0311(P1), CBU 1910(Com1), CBU 1645(DotB), CBU 0383(DNA-3-methyladenine glycosidase I), CBU 1157(lipoprotein), CBU 0008(hypothetical protein) and CBU 1869(hypothetical protein). The protein sequences were scanned for 15-mer peptides predicted to bind with high affinity to H-2 I-Ab, using a consensus approach described in [44]. Briefly, predictions were obtained from the ARB and SMM-align tool on the IEDB website [54], and all peptides were ranked according to their predicted affinity by each method. The median of the three ranks was used to select peptides for screening. A set of 117 different peptides were synthesized as crude material (A&A Labs, San Diego, CA) and used in initial screening experiments. Peptides used in immunization experiments were re-synthesized as purified material.

### Mouse procedures and immunization

C57BL/6J (B6) mice were purchased from Jackson Laboratory (Bar Harbor, ME) and bred in our facility. For screening CD4 epitopes, six-weeks old female B6 mice were subcutaneously (s.c.) vaccinated with 20 µg of each protein antigen in the context of complete Freud's adjuvant. Nine days later, CD4^+^ T cells were magnetically (MACS Miltenyibiotec, Auburn, CA) purified from spleen and lymph node preparations. For epitope and antibody linkage experiments, six-week old female B6 mice received single immunization with selected peptides in the context of incomplete Freud's adjuvant (IFA) and CpG oligonucleotide. Ten days later, primed mice were vaccinated with 2 µg PI-WCV in the context of IFA. During each vaccination, the same adjuvant without any antigen was used as sham control. Blood was collected at 7, 14, 21 and 28 days post PI-WCV vaccination. All procedures were performed under animal protocols approved by the University Laboratory Care Committee of Texas A&M University.

### ELISA

Microplates (96 well, Fisher Scientific, Pittsburgh, PA) were coated overnight at 4°C with 100 µl of 2 µg/ml antigen solution. Plates were then blocked with 200 µl of 0.5% nonfat milk for 2 h at 37°C. Individual mouse serum was diluted (1∶50) and applied to the assay wells. Specific IgG was detected using goat anti-mouse IgG HRP conjugate (Bio-Rad, Hercules, CA) and OPD peroxidase (Sigma, St. Louis, MO) by measuring OD in a Spectra Max M2 plate reader (Molecular Devices, Sunnyvale, CA).

### ELISPOT

Draining lymph nodes and spleen were pooled for each animal 9–12 days post vaccination. Then, CD4^+^ T cells were purified by CD4 (L3T4) magnetic micro-beads (Miltenyibiotec) and antigen specific IFN-γ recall responses were measured by ELISPOT. For individual mouse samples (n = 6), we incubated 2×10^5^ purified CD4^+^ T cells in triplicate with 1×10^5^ naïve lymphocytes and 1 µg purified recombinant proteins (or peptide) in a total volume of 100 µl at 37°C. The frequency of IFN-γ producing cells was quantified 20 h later by ELISPOT Readers (Autoimmun Diagnostika GmbH, Strassberg, Germany).

### H-2 I-A^b^ peptide binding assays

Peptide binding assays were performed by using purified H-2^b^ class II MHC, essentially as previously described [41]. Briefly, mouse B-cell lymphoma LB27.4 cells were utilized as sources of murine IA^b^. MHC molecules were purified by affinity chromatography using the anti-IA^b^ monoclonal antibody Y3JP. Quantitative peptide binding assays were based on the inhibition of binding of radiolabeled probe peptide ROIV (peptide 569.01, an artificial ligand with sequence YAHAAHAAHAAHAAHAA) to purified IAb molecules. Assays were performed at pH 5.5 in PBS containing 1% digitonin and in the presence of a cocktail of protease inhibitors [41]. MHC binding of the radiolabeled peptide was determined by capturing MHC-peptide complexes on antibody-coated Lumitrac 600 plates (Greiner Bio-one, Frickenhausen, Germany) and measuring bound cpm using the TopCount (Packard Instrument Co., Meriden, CT) microscintillation counter. The average 50% inhibitory concentration (IC_50_) of ROIV (peptide 569.01) for IAb was 28 nM.

### Flow cytometry and intracellular cyctokine staining (ICCS) assay

4×10^6^ splenocytes (pooled from three mice that were immunized for PI-WCV for 10 days) were either treated with 10 ug/ml peptides or 3 ug/ml *C. burnetii* Nine Mile phase I strain lysate for 18 h. Then, GolgiPlug (BD Pharmingen) was added and cells were cultured for another 6 h. Cells were incubated with the viability dye ViViD (Molecular Probes), followed by intracellular staining for PE-Cy7-anti-mouse CD3, APC-Cy7-antimouse CD4, FITC-anti-mouse-IL-2, PerCP-Cy5.5-anti-mouse-TNF-α and APC-anti-mouse IFN-γ antibodies (Biolegend, San Diego, CA) according to the manufacturer's instructions. At least 1×10^6^ events per sample were collected using a FACSAriaII (BD Biosciences) and data were analyzed with FlowJo software (TreeStar, Ashland, OR). Background values were determined from cells pulsed with DMSO only. Three independent experiments were performed for each peptide. CD40L was stained in a same manner as described above by using PE labeled anti-mouse CD154 antibody (Biolegend).

### Challenge experiments

6 to 8 week old male C57BL/6J (B6) mice, bred in the Montana State University facility were subcutaneously (s.c.) inoculated with 20 µg of each epitope or 50 µg Ova (Voigt Global Distribution, Laurence, KS) in the context of complete Freud's adjuvant, 100 µl PBS or 10^8^ genome copies of heat killed *C. burnetii* RSA 493 in 100 µl. 13 days post inoculation mice were infected intratracheally with 10^3^ genome copies of *C. burnetii* RSA 493. 14 days post infection mice were euthanized by phenobarbital anesthesia followed by exsanguination. Half of the lungs were removed, homogenized in DMEM with 10% FBS, then frozen for future DNA extraction and quantitative real time PCR.

Body weights were recorded at 2–3 day intervals after infection with *C. burnetii*. Statistical analysis was conducted using a one-way, non-parametric, ANOVA test followed by post-test Tukey analysis for experiments with more than 2 groups and experiments with 2 groups were analyzed using an unpaired t test. Differences were considered significant when *p*<0.05. For quantitative PCR, DNA was extracted from lung and spleen homogenates using a Qiagen DNeasy blood and tissue kit according to manufacturer's instructions. DNA was used as a template for quantitative real time PCR with SYBR® Green PCR Master Mix (Applied Biosystems, Foster City, CA) by quantifying *C. burnetii* genome copies from amplified *rpoS* gene copies. Determination of the number of genome copies per organ were carried out as described previously [55].

## Supporting Information

Table S1 **Complete peptide set and screening results.**
(DOC)

Figure S1 **CD4^+^ T cells recognition of PI-WCV derived H-2 I-A^b^ epitopes in IL-2 ICCS assays.** CD4^+^ T cells recognition of positive peptides identified by IFN-γ ELISPOT were tested in ICCS assay. 10 ug of each peptide was used to stimulate 2×10^6^ lymphocytes from four mice immunized with PI-WCV 10 days earlier in the context of IFA and CpG. A representative experiment of four total experiments is shown. Percentages of IL-2 producing CD4^+^ T cells following stimulation with lysed Nine Mile phase I *C. burnetii* and peptides are shown. A peptide was considered positive if the average of the individual experiments resulted in at least >1 SD above background (0.009%, Medium +DMSO).
(TIF)

Figure S2 **CD4^+^ T cells recognition of PI-WCV derived H-2 I-A^b^ epitopes in TNF-α ICCS assays.** CD4^+^ T cells recognition of positive peptides identified by IFN-γ ELISPOT were tested in ICCS assay. 10 ug of each peptide was used to stimulate 2×10^6^ lymphocytes from four mice immunized with PI-WCV 10 days earlier in the context of IFA and CpG. A representative experiment of four total experiments is shown. Percentages of TNF-α producing CD4^+^ T cells following stimulation with lysed Nine Mile Phase I *C. burnetii* and peptides are shown. A peptide was considered positive if the average of the individual experiments resulted in at least >1 SD above background (0.011%, Medium +DMSO).
(TIF)

Figure S3 **Multiparameter analysis of PI-WCV vaccination induced peptides specific CD4^+^ T cells.** CD4^+^ T cells recognition of positive peptides identified by IFN-γ ELISPOT were tested in multicolor ICCS assay as described in material and methods. 10 ug of each peptide was used to stimulate 2×10^6^ lymphocytes from four mice immunized with PI-WCV 10 days earlier in the context of IFA and CpG. Cells were gated on viable CD3^+^CD4^+^IFN-γ^+^ T cells. A representative experiment of four total experiments is shown. Percentages of TNF-α producing CD4^+^ T cells following stimulation with lysed Nine Mile phase I *C. burnetii* and peptides are shown.
(TIF)

Figure S4 **Peptide immunization does not protect from weight loss after challenge or bacterial burden.** A) Change in body weights of C57BL/6 mice immunized with either PBS alone, OVA or epitope CBU 0383_69–83_ in the context of CFA, or PI-WCV. After intratracheal infection with 10^3^ genome copies of *C. burnetii* Nine Mile phase I, body weight change was expressed as a percentage of the initial body weight prior to infection and significant differences were identified at days 7 and 10 p.i. (p<0.01). No protective effect of the epitope immunization was observed in comparison to the immunization with PBS or the irrelevant OVA epitope. Data is representative of one of two independent experiments with 4–5 mice per group. B) 14 days post infection mice were euthanized and the bacterial burden in the lung was determined by PCR. No protective effect of the epitope immunization was observed in comparison to the immunization with PBS or the irrelevant OVA epitope. In contrast, immunization with heat killed PI-WCV (positive control) resulted in significantly lower bacterial burden (p<0.01).
(TIF)
