# Supplementary material for: Identification of CD4+ T Cell Epitopes in C. burnetii Antigens Targeted by Antibody Responses
Source: PLoS One. 2011 Mar 15;6(3):e17712. doi: 10.1371/journal.pone.0017712 (PMC3057979; doi:10.1371/journal.pone.0017712)
Supplement: Table S1 — Complete peptide set and screening results. (DOC) [file pone.0017712.s001.doc]

Supplemental Table 1: Complete peptide set and screening results

| **Peptide ID** | **Sequence** | **SI^d^-rAgs^a^** | **SI^d^-WCV vaccination^b^** | **SI^d^-Infection^f^** |
| --- | --- | --- | --- | --- |
|
| CBU0383_65-79_^c,e^ | RNNYRDSFNNFDASI | 3.06 | 5.57±1.79 | 3.25±2.21 |
| CBU0383_69-83_ ^c,e^ | RDSFNNFDASIISKY | 19.33 | 5.33±3.10 | 4.37±1.32 |
| CBU0383_98-112_ | IRNKLKIQATINNAK | 2.22 | 1.07±0.51 | 0.43±0.57 |
| CBU0383_127-141_ | YIWHFVDGHPIQNQW | 16.22 | 1.33±1.43 | 0.79±0.28 |
| CBU1157_61-75_ | RGPWRYIRSFPILAS | 7.17 | 1.87±1.67 | 0.57±0.34 |
| CBU1157_63-77_ ^c,e^ | PWRYIRSFPILASSG | 7.63 | 6.80±1.25 | 4.23±1.73 |
| CBU1157_108-122_ ^c^ | LSLMLNYPNSADRYY | 3.08 | 4.83±2.44 | 1.78±0.45 |
| CBU1157_177-191_^c,e^ | DLRYHAPIYGAVHPR | 3.58 | 3.67±1.53 | 3.83±1.56 |
| CBU1157_179-193_ | RYHAPIYGAVHPRWL | 4.37 | 1.97±0.75 | 2.37±1.05 |
| CBU1645_10-24_ | RYPNEPSRFEPKHID | 2.38 | 2.03±1.27 | 1.56±1.29 |
| CBU1645_138-152_ | DLPAAIVDAIAPQEG | 3 | 0.80±0.62 | 0.67±0.34 |
| CBU1645_194-208_ | YVYDSLTTPTASVCQ | 6 | 0.67±0.25 | 0.43±0.17 |
| CBU1645_196-210_ ^c,e^ | YDSLTTPTASVCQSE | 6.67 | 4.13±1.01 | 3.52±1.47 |
| CBU1645_270-284_ ^c,e^ | IRRLVGSFPAEERIG | 2.7 | 3.10±1.15 | 1.53±0.74 |
| CBU1645_272-286_ ^c,e^ | RLVGSFPAEERIGRT | 3.2 | 3.50±0.96 | 1.78±0.98 |
| CBU1910_43-57_ ^c,e^ | VHHYLVNHPEVLVEA | 15.9 | 22.73±12.51 | 28.27±9.54 |
| CBU1910_45-59_ ^c,e^ | HYLVNHPEVLVEASQ | 17.1 | 23.77±13.0 | 36.23±15.76 |
| CBU1910_79-93_ | ENAKKLFNDPASPVA | 5.6 | 0.93±0.84 | 0.93±0.35 |
| CBU1910_81-95_ | AKKLFNDPASPVAGN | 4.8 | 0.63±0.31 | 0.79±0.44 |
| CBU1910_83-97_ ^c,e^ | KLFNDPASPVAGNPH | 3.8 | 3.67±1.12 | 3.81±1.31 |
| CBU0008_8-22_ | QKQQKAVSDSPALSN | 0.3 | N.A | N.A |
| CBU0008_10-24_ | QQKAVSDSPALSNFF | 0.7 | N.A | N.A |
| CBU0008_13-27_ | AVSDSPALSNFFISV | 0.5 | N.A | N.A |
| CBU0008_26-40_ | SVIFKSVTSIPIYLS | 0.5 | N.A | N.A |
| CBU0008_28-42_ | IFKSVTSIPIYLSSN | 1.3 | N.A | N.A |
| CBU0008_30-44_ | KSVTSIPIYLSSNWR | 1.2 | N.A | N.A |
| CBU0008_35-49_ | IPIYLSSNWRTTNPP | 0.8 | N.A | N.A |
| CBU0008_39-53_ | LSSNWRTTNPPKPQP | 1 | N.A | N.A |
| CBU0008_41-55_ | SNWRTTNPPKPQPLK | 1.5 | N.A | N.A |
| CBU0008_43-57_ | WRTTNPPKPQPLKLT | 1.1 | N.A | N.A |
| CBU0008_45-59_ | TTNPPKPQPLKLTQG | 0.4 | N.A | N.A |
| CBU0008_53-67_ | PLKLTQGRATFVSPV | 1.3 | N.A | N.A |
| CBU0311_16-30_ | ALCCLASAAFAGGPD | 2 | N.A | N.A |
| CBU0311_18-32_ | CCLASAAFAGGPDIP | 1.1 | N.A | N.A |
| CBU0311_21-35_ | ASAAFAGGPDIPMID | 1.1 | N.A | N.A |
| CBU0311_44-58_ | GFGYKSYTYDQVGTV | 1.4 | N.A | N.A |
| CBU0311_47-61_ | YKSYTYDQVGTVTVT | 1.6 | N.A | N.A |
| CBU0311_49-63_ | SYTYDQVGTVTVTTN | 1.1 | N.A | N.A |
| CBU0311_63-77_ | NGGTVLSVLHPVSAS | 1.9 | N.A | N.A |
| CBU0311_65-79_ | GTVLSVLHPVSASIT | 1.5 | N.A | N.A |
| CBU0311_68-82_ | LSVLHPVSASITQFG | 2 | N.A | N.A |
| CBU0311_73-87_ | PVSASITQFGPVGEL | 0.9 | N.A | N.A |
| CBU0311_86-100_ | ELGYTFASDWWIAGV | 1.6 | N.A | N.A |
| CBU0311_89-103_ | YTFASDWWIAGVKAQ | 1.1 | N.A | N.A |
| CBU0311_92-106_ | ASDWWIAGVKAQYQY | 1.9 | N.A | N.A |
| CBU0311_109-123_ | VRSVHIMDAPLVGSN | 1.3 | N.A | N.A |
| CBU0311_111-125_ | SVHIMDAPLVGSNYS | 1.6 | N.A | N.A |
| CBU0311_114-128_ | IMDAPLVGSNYSYRT | 1.3 | N.A | N.A |
| CBU0311_133-147_ | HLTAMLLAGIKVNEA | 1.4 | N.A | N.A |
| CBU0311_139-153_ | LAGIKVNEANAVYLE | 1.3 | N.A | N.A |
| CBU0311_148-162_ | NAVYLEAGYSTVWGK | 1.9 | N.A | N.A |
| CBU0311_160-174_ | WGKTTLFGPGPVAVS | 1.1 | N.A | N.A |
| CBU0311_162-176_ | KTTLFGPGPVAVSMK | 1.2 | N.A | N.A |
| CBU0311_164-178_ | TLFGPGPVAVSMKNR | 1.3 | N.A | N.A |
| CBU0311_199-213_ | DLSYDYALYRSKSNS | 1.7 | N.A | N.A |
| CBU0311_204-218_ | YALYRSKSNSVTLSS | 1.4 | N.A | N.A |
| CBU0311_207-221_ | YRSKSNSVTLSSATA | 1.4 | N.A | N.A |
| CBU0311_209-223_ | SKSNSVTLSSATASA | 1.8 | N.A | N.A |
| CBU0311_212-226_ | NSVTLSSATASAEGT | 1.1 | N.A | N.A |
| CBU0311_229-243_ | GVSGTVQNPKRVAIN | 2 | N.A | N.A |
| CBU0311_238-252_ | KRVAINGITATVNYL | 1.2 | N.A | N.A |
| CBU0383_100-114_ | NKLKIQATINNAKAF | 1.4 | N.A | N.A |
| CBU0383_170-184_ | GSTICYAFMQAVGMV | 1 | N.A | N.A |
| CBU0383_172-186_ | TICYAFMQAVGMVND | 1.8 | N.A | N.A |
| CBU0383_191-205_ | CFRYEEIKNLPILLS | 0.8 | N.A | N.A |
| CBU1157_21-35_ | GYAFKNLGPYFKRAG | 0.7 | N.A | N.A |
| CBU1157_91-105_ | PEGVYKIVGFNPASR | 0.3 | N.A | N.A |
| CBU1157_93-107_ | GVYKIVGFNPASRFD | 1 | N.A | N.A |
| CBU11571_42-156_ | ASIGCIPIGNKAIEQ | 0.5 | N.A | N.A |
| CBU1157_153-167_ | AIEQLFVLAYLVGER | 0.8 | N.A | N.A |
| CBU1157_182-196_ | APIYGAVHPRWLPQL | 0.4 | N.A | N.A |
| CBU1157_184-198_ | IYGAVHPRWLPQLYA | 0.4 | N.A | N.A |
| CBU1645_4-18_ | MIEKSQRYPNEPSRF | 0.6 | N.A | N.A |
| CBU1645_7-21_ | KSQRYPNEPSRFEPK | 0.7 | N.A | N.A |
| CBU1645_12-26_ | PNEPSRFEPKHIDDL | 1.2 | N.A | N.A |
| CBU1645_37-51_ | DITIQTGRAVLAEVY | 1 | N.A | N.A |
| CBU1645_120-134_ | IQITIRAIPAEPPLL | 0.6 | N.A | N.A |
| CBU1645_123-137_ | TIRAIPAEPPLLSKL | 0.9 | N.A | N.A |
| CBU1645_126-140_ | AIPAEPPLLSKLDLP | 0.5 | N.A | N.A |
| CBU1645_133-147_ | LLSKLDLPAAIVDAI | 0.9 | N.A | N.A |
| CBU1645_140-154_ | PAAIVDAIAPQEGVV | 0.7 | N.A | N.A |
| CBU1645_142-156_ | AIVDAIAPQEGVVYV | 0.6 | N.A | N.A |
| CBU1645_184-198_ | KILTYEAPIEYVYDS | 0.8 | N.A | N.A |
| CBU1645_211-225_ | IPRHLNSFAAGVRNA | 0.9 | N.A | N.A |
| CBU1645_224-238_ | NALRRKPHAILVGEA | 0.7 | N.A | N.A |
| CBU1645_248-262_ | LEAALTGHPVYTTLH | 0.6 | N.A | N.A |
| CBU1645_251-265_ | ALTGHPVYTTLHSSG | 0.5 | N.A | N.A |
| CBU1645_255-269_ | HPVYTTLHSSGVAET | 0.7 | N.A | N.A |
| CBU1645_268-282_ | ETIRRLVGSFPAEER | 1 | N.A | N.A |
| CBU1645_294-308_ | RLIIWQRLVPSVDGK | 0.7 | N.A | N.A |
| CBU1645_352-366_ | DVEAKFKGGLISERL | 0.6 | N.A | N.A |
| CBU1869_100-114_ | HSGQFIVLPLPITKK | 1.8 | N.A | N.A |
| CBU1869_102-116_ | GQFIVLPLPITKKQL | 1.6 | N.A | N.A |
| CBU1869_121-135_ | NYEVIAKKYPHVNAW | 0.6 | N.A | N.A |
| CBU1869_123-137_ | EVIAKKYPHVNAWQI | 1.9 | N.A | N.A |
| CBU1869_125-139_ | IAKKYPHVNAWQILD | 1.5 | N.A | N.A |
| CBU1869_127-141_ | KKYPHVNAWQILDFP | 1.6 | N.A | N.A |
| CBU1869_131-145_ | HVNAWQILDFPQEIK | 1.5 | N.A | N.A |
| CBU1869_148-162_ | RNRYRLVFTQQLKDG | 1.7 | N.A | N.A |
| CBU1910_13-27_ | LAGTLTAGVAIAAPS | 0.7 | N.A | N.A |
| CBU1910_16-30_ | TLTAGVAIAAPSQFS | 0.6 | N.A | N.A |
| CBU1910_19-33_ | AGVAIAAPSQFSFSP | 0.6 | N.A | N.A |
| CBU1910_21-35_ | VAIAAPSQFSFSPQQ | 1.1 | N.A | N.A |
| CBU1910_23-37_ | IAAPSQFSFSPQQVK | 1.4 | N.A | N.A |
| CBU1910_25-39_ | APSQFSFSPQQVKDI | 1 | N.A | N.A |
| CBU1910_28-42_ | QFSFSPQQVKDIQSI | 0.6 | N.A | N.A |
| CBU1910_54-68_ | LVEASQALQKKTEAQ | 0.7 | N.A | N.A |
| CBU1910_86-100_ | NDPASPVAGNPHGNV | 1 | N.A | N.A |
| CBU1910_90-104_ | SPVAGNPHGNVTLVE | 0.4 | N.A | N.A |
| CBU1910_143-157_ | QSQYAAKVSLAAAKQ | 0.8 | N.A | N.A |
| CBU1910_145-159_ | QYAAKVSLAAAKQGK | 0.8 | N.A | N.A |
| CBU1910_212-226_ | LAQSLQLAGTPTFVI | 0.7 | N.A | N.A |
| CBU1910_214-228_ | QSLQLAGTPTFVIGN | 1.3 | N.A | N.A |
| CBU1910_216-230_ | LQLAGTPTFVIGNKA | 0.5 | N.A | N.A |
| CBU1910_227-241_ | GNKALTKFGFIPGAT | 0.6 | N.A | N.A |
| CBU1910_229-243_ | KALTKFGFIPGATSQ | 0.7 | N.A | N.A |
| CBU1910_232-246_ | TKFGFIPGATSQQNL | 0.7 | N.A | N.A |

^a^ Mice were subcutaneously vaccinated with 20ug rAgs/mouse(CFA) for 9-12 days. Peptide at 1ug/ml. Average of two independent experiment (n=5).

^b^ Mice were subcutaneously vaccinated with 10ug PI-WCV/mouse(IFA+CpG) for 10 days. Peptide at 1ug/ml. Three Independent experiment (n=6).

^c^ Elicit positive positive signal (>20 SFC/10^6^ cells, SI>2 and a *p*<0.05) in both rAgs and PI-WCV vaccinated B6 mice.

^d^ SI was calculated as the SFC of experimental peptide divided by SFC of medium background.

^e^. Peptides were partially overlapping with other peptides.

^f^ Mice were intratracheally infected using 10^6^ *C. burnetii* RSA439 strain for 10 days. Peptide at 1ug/ml. Three independent experiment (n=4).
