# Supplementary figures and images for: Identification of CD4+ T Cell Epitopes in C. burnetii Antigens Targeted by Antibody Responses
Source: PLoS One. 2011 Mar 15;6(3):e17712. doi: 10.1371/journal.pone.0017712 (PMC3057979; doi:10.1371/journal.pone.0017712)

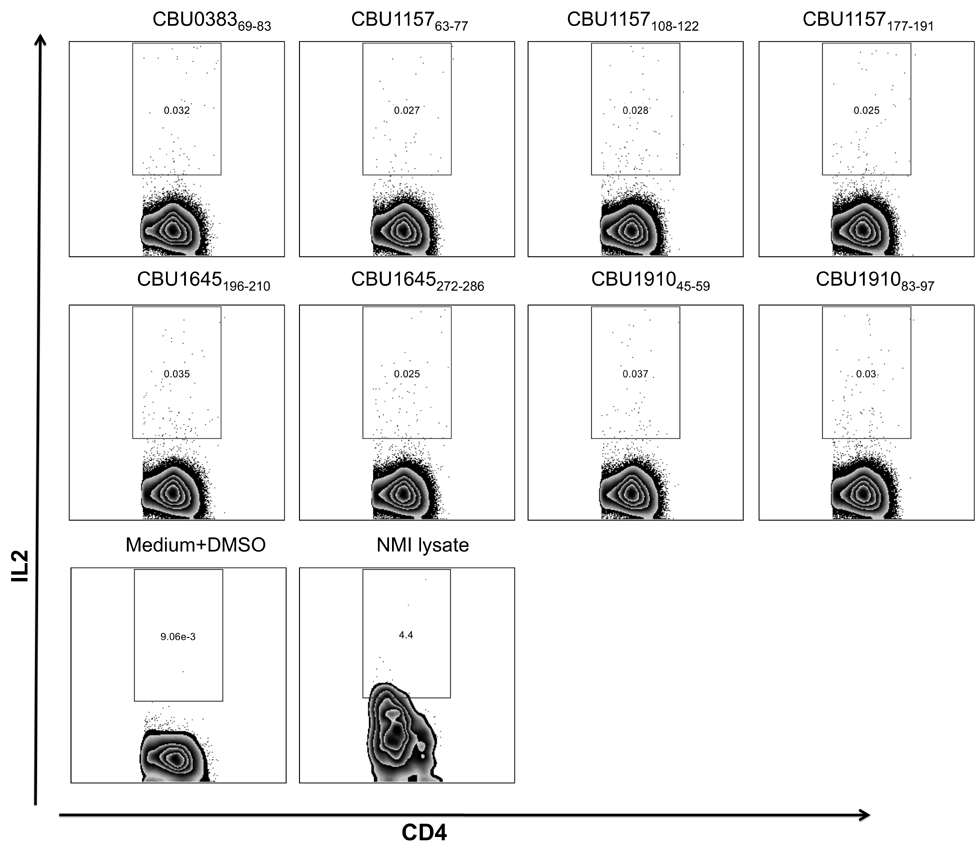

Supplement: Figure S1 — CD4+ T cells recognition of PI-WCV derived H-2 I-Ab epitopes in IL-2 ICCS assays. CD4+ T cells recognition of positive peptides identified by IFN-γ ELISPOT were tested in ICCS assay. 10 ug of each peptide was used to stimulate 2×10^6^ lymphocytes from four mice immunized with PI-WCV 10 days earlier in the context of IFA and CpG. A representative experiment of four total experiments is shown. Percentages of IL-2 producing CD4+ T cells following stimulation with lysed Nine Mile phase I C. burnetii and peptides are shown. A peptide was considered positive if the average of the individual experiments resulted in at least >1 SD above background (0.009%, Medium +DMSO). (TIF) [file pone.0017712.s002.tif]

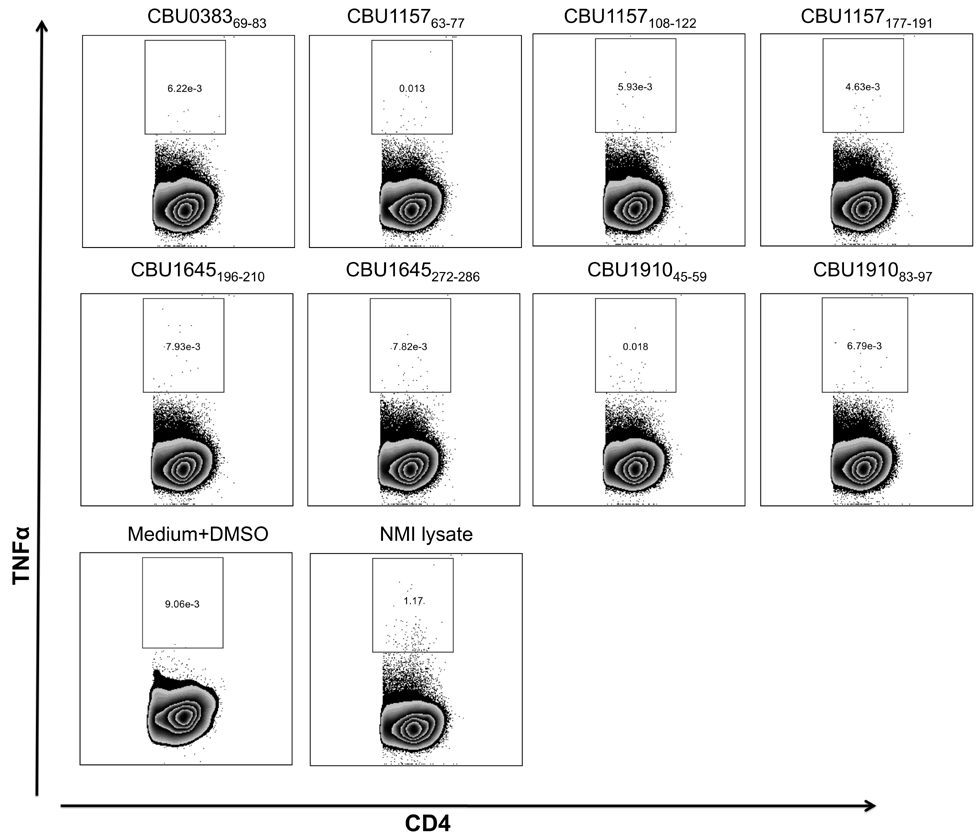

Supplement: Figure S2 — CD4+ T cells recognition of PI-WCV derived H-2 I-Ab epitopes in TNF-α ICCS assays. CD4+ T cells recognition of positive peptides identified by IFN-γ ELISPOT were tested in ICCS assay. 10 ug of each peptide was used to stimulate 2×10^6^ lymphocytes from four mice immunized with PI-WCV 10 days earlier in the context of IFA and CpG. A representative experiment of four total experiments is shown. Percentages of TNF-α producing CD4+ T cells following stimulation with lysed Nine Mile Phase I C. burnetii and peptides are shown. A peptide was considered positive if the average of the individual experiments resulted in at least >1 SD above background (0.011%, Medium +DMSO). (TIF) [file pone.0017712.s003.tif]

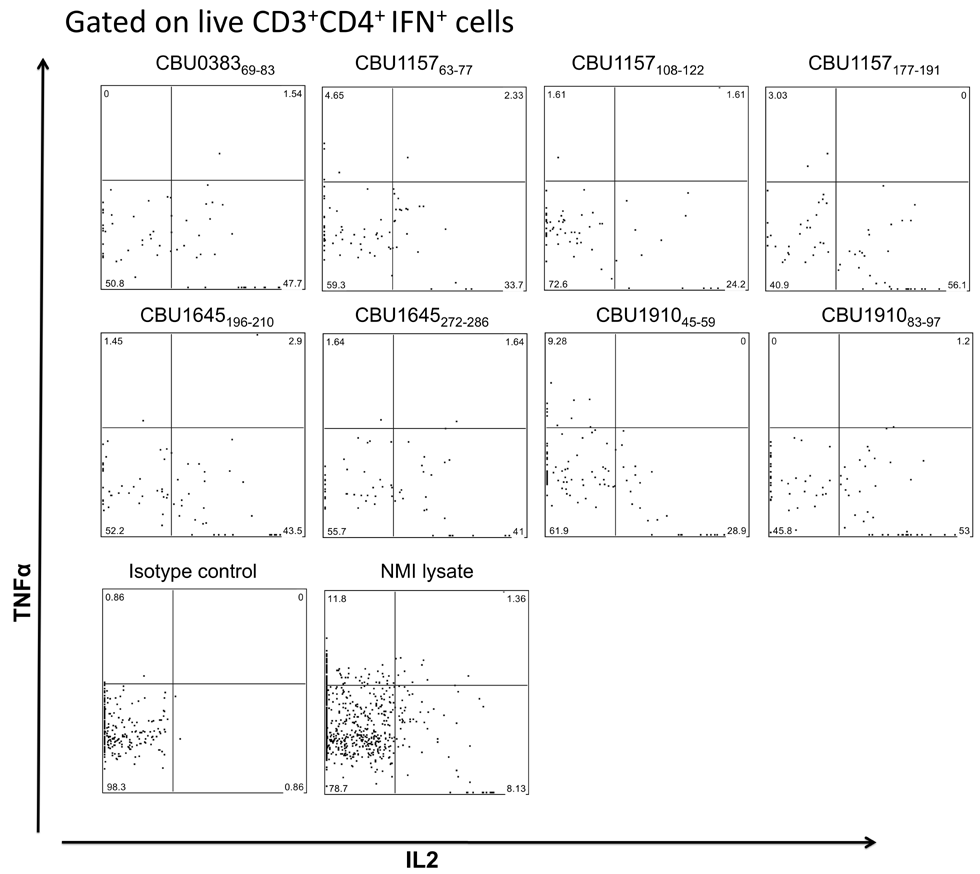

Supplement: Figure S3 — Multiparameter analysis of PI-WCV vaccination induced peptides specific CD4+ T cells. CD4+ T cells recognition of positive peptides identified by IFN-γ ELISPOT were tested in multicolor ICCS assay as described in material and methods. 10 ug of each peptide was used to stimulate 2×10^6^ lymphocytes from four mice immunized with PI-WCV 10 days earlier in the context of IFA and CpG. Cells were gated on viable CD3+CD4+IFN-γ+ T cells. A representative experiment of four total experiments is shown. Percentages of TNF-α producing CD4+ T cells following stimulation with lysed Nine Mile phase I C. burnetii and peptides are shown. (TIF) [file pone.0017712.s004.tif]

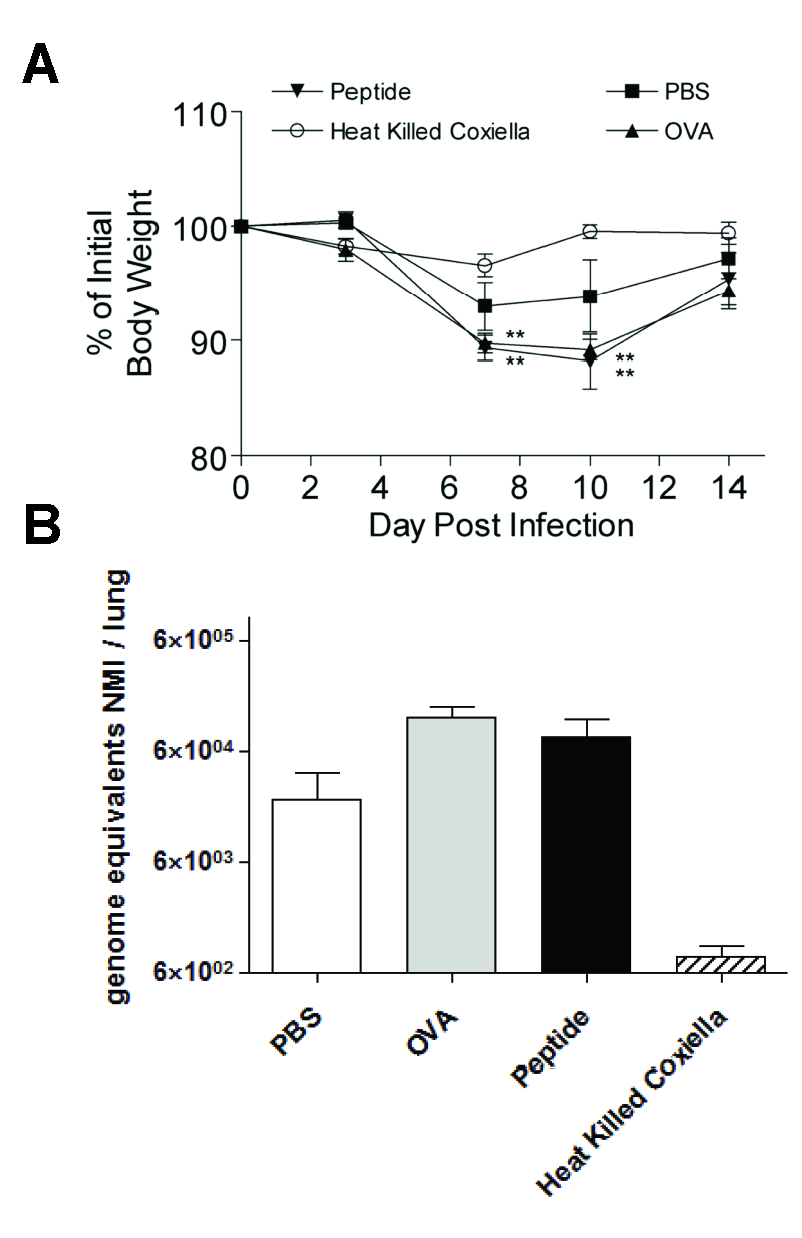

Supplement: Figure S4 — Peptide immunization does not protect from weight loss after challenge or bacterial burden. A) Change in body weights of C57BL/6 mice immunized with either PBS alone, OVA or epitope CBU 038369–83 in the context of CFA, or PI-WCV. After intratracheal infection with 103 genome copies of C. burnetii Nine Mile phase I, body weight change was expressed as a percentage of the initial body weight prior to infection and significant differences were identified at days 7 and 10 p.i. (p<0.01). No protective effect of the epitope immunization was observed in comparison to the immunization with PBS or the irrelevant OVA epitope. Data is representative of one of two independent experiments with 4–5 mice per group. B) 14 days post infection mice were euthanized and the bacterial burden in the lung was determined by PCR. No protective effect of the epitope immunization was observed in comparison to the immunization with PBS or the irrelevant OVA epitope. In contrast, immunization with heat killed PI-WCV (positive control) resulted in significantly lower bacterial burden (p<0.01). (TIF) [file pone.0017712.s005.tif]
